# Knowledge and perception of medical students regarding remote-access thyroidectomy in Tabuk: the effects of extensive counseling—an interventional study

**DOI:** 10.3389/fsurg.2024.1428046

**Published:** 2024-09-18

**Authors:** Hyder Mirghani, Amirah Alhowiti

**Affiliations:** ^1^ Department of Internal Medicine, Faculty of Medicine, University of Tabuk, Tabuk, Saudi Arabia; ^2^ Department of Family and Community Medicine, Faculty of Medicine, University of Tabuk, Tabuk, Saudi Arabia

**Keywords:** extensive counseling, operation choice, medical students, Tabuk, Saudi Arabia

## Abstract

**Introduction:**

Remote-access scarless thyroidectomies are relatively new surgical procedures, and their uptake for cosmetic concerns is rapidly evolving. However, demographic factors, previous thyroid surgery, and culture substantially influence the patient's choice. This is the first study to assess the extensive counseling effect on the patient's preference for remote-access thyroidectomies compared to conventional transcervical approaches. We aimed to assess the same among medical students at the University of Tabuk, Saudi Arabia.

**Methods:**

This interventional study was conducted from December 2023 to March 2024. A structured questionnaire was used to gather information about demographic factors, knowledge, and perception of the medical students regarding remote-access thyroidectomies before and after a slide presentation.

**Results:**

Of 394 medical students (age 22.65 ± 1.62 years), 53.8% were women. Initially, the majority of the students preferred remote-access thyroidectomy over the conventional approach (85.3% vs. 14.7%, respectively); however, a substantial change toward the cervical approach was evident after extensive counseling [odds ratio, 2.59; 95% confidence interval (CI), 1.58–4.27; and *P*-value <0.00]. Knowledge regarding remote-access thyroidectomy was poor (26.9%). The students were anxious regarding postoperative complications (3.22 ± 1.59/5), thyroid scar appearance (3.28 ± 1.36/5), and postoperative pain (3.17 ± 1.38/5). Concerns regarding body satisfaction and cost were lower.

**Conclusion:**

Medical students at the University of Tabuk, Saudi Arabia, demonstrated low knowledge. The strong preference for remote-access thyroidectomy over transcervical thyroidectomy shifted substantially toward the conventional approach after counseling. The main factors were thyroid scar appearance, pain, and complications. Further larger-scale studies involving the general public and assessing the effect of extensive counseling on surgical choice are needed.

## Introduction

Thyroid nodules (TNs) are common conditions, and their diagnosis is steadily increasing in some countries due to the development of modern imaging technologies (1). As a result, the rates of lobectomy and total thyroidectomy have increased, mirroring the higher diagnosis of TNs. Thyroidectomy is increasingly performed on an outpatient basis and has an excellent safety profile (2). In addition to nodules, thyroidectomy is indicated for malignancy, large goiters with pressure symptoms, retrosternal extension, and severe ophthalmic manifestations of Graves’ disease. Other indications include failure and contraindication to other therapeutic modalities (3). Remote-access thyroidectomy was introduced in 1997 for limited thyroid disease and has evolved to include many approaches such as axillary access, anterior chest, areolar breast, and minimal neck incision. The extra-cervical (remote) approaches are scarless and have shown similar postoperative complications, albeit with more postoperative pain (4). In the last two decades, remote-access thyroidectomy has gained popularity and has increasingly advanced. The transoral endoscopic thyroidectomy vestibular approach (TOETVA) adopted an oral incision to avoid scarring in the neck and improve cosmesis (5). The paradigm shift is the development of several techniques to approach the thyroid gland (through four incisions in the axilla and breast, an axillary incision, an incision through the oral vestibule, or incisions behind the ear, which are known as the bilateral axillo-breast approach, transaxillary technique, vestibular approach, and retro-auricular technique, respectively (6, 7). The indications and contraindications for remote-access thyroidectomy remain to be elucidated. However, favorable patient characteristics for remote-access thyroidectomy include motivation to avoid a neck scar, symptomatic benign nodules ≥6 cm, thyroid diameter ≤10 cm, thyroid volume ≤45 cm on ultrasound, and Graves’ disease (euthyroid if possible). Additional factors include symptomatic Hashimoto's thyroiditis and differentiated thyroid cancer <3 cm without extrathyroidal or lymph node extension preoperatively. Non-favorable features include substernal extension, previous neck and chin surgery, and previous neck irradiation (4).

Remote-access thyroidectomy is gaining popularity and is spreading all over the world, including in Asia (9, 10), Europe (11), and North America (12). Saudi Arabia is no exception, and remote-access scarless thyroidectomy is increasingly being performed due to cosmetic concerns, mirroring the high rate of thyroid nodule diagnoses. Although these procedures are still in their initial stage in the Kingdom of Saudi Arabia, they are expected to increase shortly (13).

Cosmetic issues beyond the limitations of conventional surgery have pushed surgeons. Therefore, the remote-access endoscopic thyroidectomy is expected to increase shortly. The cosmetic outcomes (avoidance of a visible scar) come at the expense of long operative times, extended postoperative hospital stays, high costs, the need for specialized surgical training, and a steep learning curve (8).

Thyroidectomy (in particular for cancer) can be extremely distressing for patients; therefore, understanding patients' attitudes toward remote-access thyroidectomy is vital for shared decision-making and for enabling health authorities to plan what type of surgery is needed (14). Medical students are an important segment of the community; they are the future doctors and their knowledge will be reflected in the whole community. Therefore, we assessed the knowledge and attitudes of clinical-phase medical students at the University of Tabuk toward remote-access thyroid surgery. In addition, we investigated the effects of slideshow counseling on students' preferences regarding the route of thyroidectomy.

## Methods

This interventional study was conducted among the clinical-phase medical students at the Faculty of Medicine, University of Tabuk, Saudi Arabia, from December 2023 to March 2024. Data were collected using a structured web-based questionnaire, which consisted of two parts. The first part gathered demographic data, including class, sex, and age. The second part inquired about students’ knowledge and preference regarding the following thyroidectomy approaches: transcervical, transoral, postauricular, and breast axillary approaches. The knowledge questions were based on the operative time, postoperative bleeding, cosmetic outcomes, and quality of life of different procedures. The questionnaire was adapted from the previous literature and approved by a senior oncology surgeon and the two authors. The second author distributed the questionnaire in a classroom, where the students responded to it. A slideshow presentation was given, featuring photographs detailing the stages of remote-access thyroidectomy and conventional transcervical thyroidectomy. In addition, the complications of both procedures, including infection, antibiotic use, and lip paresthesia, were discussed. Furthermore, the evolution of transcervical surgery and the excellent cosmetic scar were discussed with the students.

The students were invited to respond again to the perception section of the questionnaire after the presentation and discussion. Poor knowledge is to be expected at this student level, given the relatively new and specialized nature of the procedure. The students' knowledge was assessed at baseline as an entrance to the main objective of the current study, which was to evaluate the influence of counseling on the students' choice of thyroidectomy operation.

A Likert scale from 1 (strongly disagree) to 5 (strongly agree) was used to assess the knowledge and perception of the medical students regarding different types of thyroidectomy. Randomly chosen images for each remote surgery and scarless surgery were included in the slideshow. In addition, the operative and postoperative complications of the different approaches were discussed with the students. Body image perception was assessed by Park et al. (15), and the complications were adapted from two previous studies (16, 17).

### Inclusion criteria

All clinical-phase medical students at the University of Tabuk, Saudi Arabia, were included.

### Exclusion criteria

Preparatory and basic-phase medical students at the University of Tabuk, Saudi Arabia, were excluded because of expected extremely poor knowledge.

### Sample size

The sample size was calculated using the following sample size calculator: https://www.calculator.net/sample-size-calculator.html?type=1&cl=95&ci=5&pp=50&ps=340&x=79&y=26 (18). With a 95% confidence interval (CI), a 5% margin of error, and a population proportion of 50%, the sample size was calculated to be 203 students. However, the sample size was increased to 394 to minimize the error and to address the expected low response after the intervention. Therefore, the response rate was 92.3% (394/426 = 92.3%).

### Intervention

After the students filled out the questionnaire, the second author presented a slideshow including photographs of transcervical thyroidectomy and different remote-access endoscopic thyroidectomies. A second round of responses for the perception section of the questionnaire was collected after 2 weeks.

### Outcome measures

The outcome measures were the students’ knowledge and perception of different routes to thyroidectomy. In addition, the influence of counseling (by slide presentation) on the students' preferences was investigated.

### Ethical issues

All students were informed about the purpose of the research before responding to the questionnaire; then, a question followed: Do you agree to participate in this study? Those who selected “disagree” were blocked, and their responses were not included. The ethical committee of the University of Tabuk approved the research (UT-266-114-2023, dated 13 March 2023). The recruitment period ranged from 1 December 2023 to 1 March 2024.

The research was conducted in strict accordance with the Declaration of Helsinki.

Participants' anonymity was ensured by assigning each participant a code number for analysis. The participants were informed that their participation was voluntary, that no incentives were provided, and that their information would not be shared.

### Statistical analysis

Web-based Excel data were downloaded and transferred to the Statistical Package for Social Sciences (IBM, SPSS, version 20, New York, NY, USA). Continuous data were presented as mean ± SD, and categorical data were presented as percentages. The chi-squared test was used to assess the effect of extensive counseling on students' preference for thyroidectomy. A *P*-value of <0.05 was considered significant.

## Results

The study included 394 clinical-phase medical students with a mean age of 22.65 ± 1.62 years, of whom 46.2% were women. The majority of the students preferred lobectomy over conventional transcervical thyroidectomy (85.3% vs. 14.7%). This result suggests that the students preferred to take the minimal risk associated with a second thyroidectomy (completion thyroidectomy) for differentiated thyroid carcinoma. Notably, the choice of conventional thyroidectomy increased at the expense of remote-access surgery following a slideshow, with a significant statistical difference (*P*-value <0.001; odds ratio, 2.59; and 95% CI, 1.58–4.27) (Tables 1, 2).

**Table 1:** Basic characteristics of clinical-phase medical students, University of Tabuk, Saudi Arabia.

Characteristics	No (%)
Age (range 20–33 years), mean ± SD	22.65 ± 1.62
Gender	
Men	212 (53.8%)
Women	182 (46.2%)
Previous thyroid surgery	022 (5.6%)
Students' preference regarding operation type	
Lobectomy	333 (84.5%)
Total thyroidectomy	61 (15.5%)
Preferred type of surgery (Remote-access vs. conventional) before counseling	
Conventional transcervical thyroidectomy	58 (14.7%)
Remote-access surgery	336 (85.3%)
Preferred type of surgery (Remote-access vs. conventional) after counseling	
Conventional transcervical thyroidectomy	122 (31.0%)
Remote-access surgery	272 (69.0%)
Knowledge	
Good	106 (26.5%)
Poor	288 (73.1%)

**Table 2:** Effect of counseling on choice of operation (remote vs. conventional).

Character	Before counseling	After counseling	*P*-value	Odds ratio	95% CI
Lobectomy	336 (85.3%)	272 (69.0%)	<0.001	2.59	1.58–4.27
Transcervical (74)	58 (14.7%)	122 (31.0%)			

Although operative and postoperative outcomes varied substantially across countries, the students were not right in their perceptions; they thought that remote-access thyroidectomy was better in terms of operation time (54.1% vs. 45.9%), postoperative pain (55.3% vs. 44.7%), and operative blood loss (55.3% vs. 44.7%). Other components of the medical student knowledge regarding remote-access and conventional thyroidectomy are illustrated in Table 3.

**Table 3:** Medical student knowledge of thyroid surgery complications and outcomes.

Which of the following is better?	Conventional thyroidectomy	Remote-access thyroidectomy
Operation time	181 (45.9%)	213 (54.1%)
Postoperative pain	176 (44.7%)	218 (55.3%)
Operative blood loss	169 (44.8%)	218 (55.3%)
Quality of life	130 (33.2%)	263 (66.8%)
Patient satisfaction	135 (34.3%)	259 (65.7%)
Postoperative hospital stay	168 (42.6%)	226 (57.4%)

The students were most concerned about postoperative complications (3.22 ± 1.59/5), thyroid scar appearance (3.28 ± 1.36/5), and postoperative pain (3.17 ± 1.38/5). They were less concerned regarding difficulty looking at self after the operation (2.24 ± 1.30/5), body damage following the operation (2.43 ± 1.23/5), feeling less attractive (2.45 ± 1.29/5), body satisfaction (2.56 ± 1.31/5), and cost (2.71 ± 1.36/5). Table 4 presents the students' concerns regarding thyroidectomy.

**Table 4:** Perception of medical students, University of Tabuk, regarding remote endoscopic thyroidectomy.

Characteristics (Likert scale, 1–5 points)	Mean	Std. deviation
Importance of thyroid scar appearance	3.22	1.59
Would you be less satisfied with your body after the operation?	2.56	1.31
Would you feel that the operation damaged your body?	2.43	1.23
Would you feel less attractive after the operation?	2.45	1.29
Would you find it difficult to look at yourself after the operation?	2.24	1.30
Postoperative pain concerns	3.17	1.38
Scar concerns	3.02	1.40
Postoperative complications concerns	3.28	1.44
Cost concerns	2.71	1.36

**Figure 1:**
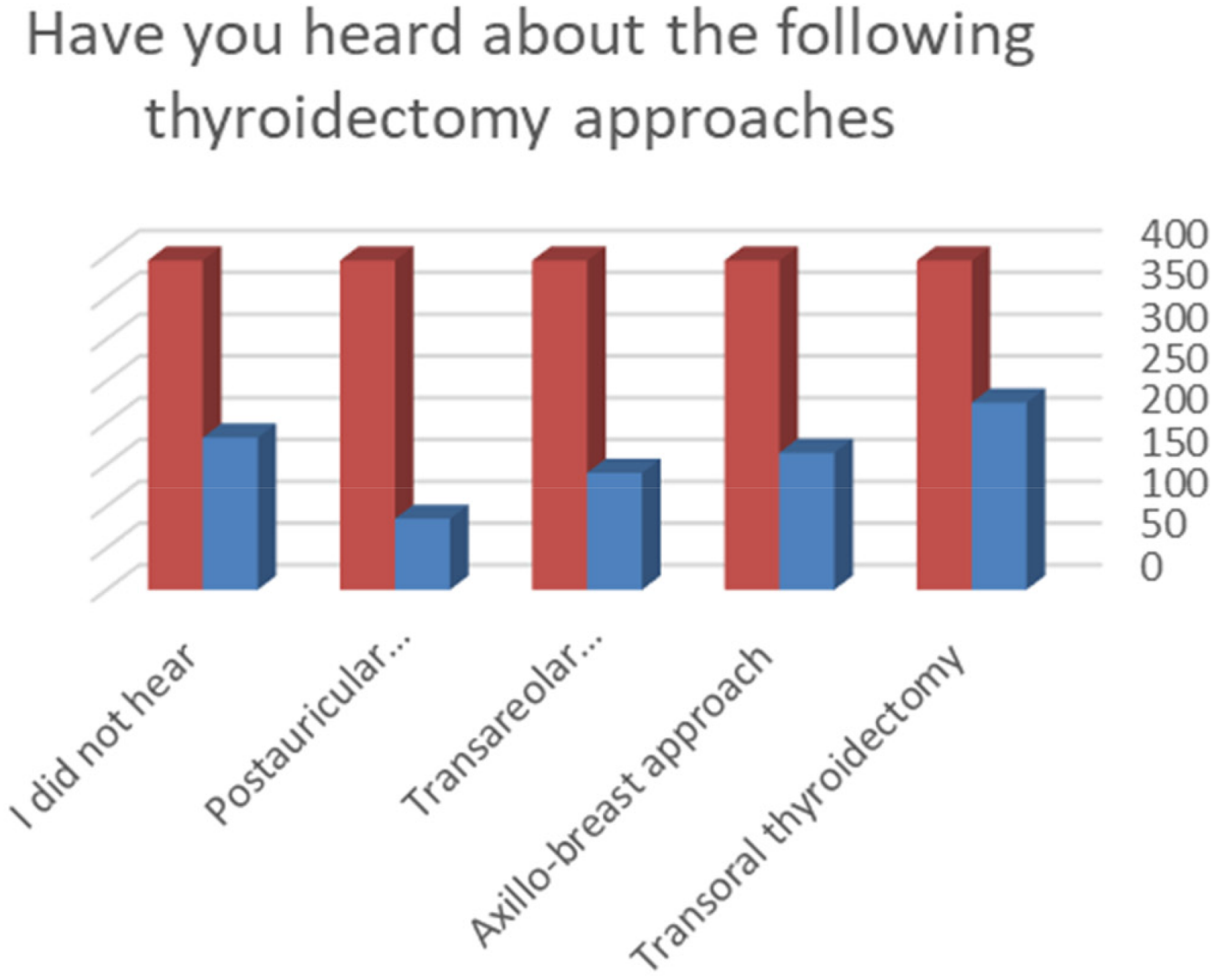
Medical students in Tabuk, Saudi Arabia knowledge about different remote thyroidectomy procedures.

Interestingly, 56.8%, 41.6%, 35.6%, and 21.6% of the students had heard about transoral endoscopic thyroidectomy vestibular approach, postauricular thyroidectomy, transareolar thyroidectomy, and axillo-breast thyroidectomy, respectively (Figure 1).

## Discussion

In this study, despite the poor knowledge observed among medical students, they preferred remote-access thyroidectomy. However, a statistically significant shift toward a more conventional transcervical approach was evident after a slideshow detailing the different thyroid surgeries and operative and postoperative complications. A previous study conducted in the United States in 966 patients (the majority being women, mean age 40 ± 17 years) found that remote-access scarless thyroidectomy was the preferred approach (19). In the current data, remote-access thyroidectomy was chosen by 85.3% of medical students, supporting the previous observation. The current findings contrast with many previous studies. Wu et al. from Canada recently published a study and found no difference in patient choice between conventional transcervical and transoral thyroidectomy (20). The authors observed that the choice of operation was driven mainly by gender and ethnicity (with white women preferring the remote approach). An additional plausible explanation for the contradicting results is that the authors assessed only transcervical vs. transoral approaches, while we adopted a broader perspective by including four types of remote procedures. Similarly, Linos et al. (21) from Greece conducted a study over a 10-year period and found a preference for the remote approach, consistent with the current results; the authors explained their findings by citing the cost, pain, and long duration of the robotic approach. Importantly, the authors of the above study compared transcervical thyroidectomy with the transaxillary approach. Patients from the United States were less concerned about scar appearance and did not hide them, according to Best et al. However, women with long scars may do so (22). The findings of Best et al. did not support a previous study published in the United Kingdom, which showed no correlation between scar length and satisfaction (23). An interesting finding was added to the factors mediating the choice of operation: Arora et al. (24) hypothesized that the patient's psychological response to the diagnosis of cancer, rather than a benign disease, is the game player. Accordingly, patients diagnosed with differentiated thyroid cancer were less satisfied with their scar appearance compared to their counterparts with benign conditions. In the present study, cosmetic concerns significantly influenced patients’ decision-making when choosing between different thyroidectomy procedures. Similarly, Sukpanich et al. (25) observed that younger patients preferred the remote approach. Importantly, the mean age of the current sample was 22.65 ± 1.62 years, which may have skewed the findings toward a preference for remote operations. Interestingly, not only the presence of scars matters but also their site and length; shorter, lower scars are preferred over longer scars. Of note is the fact that the presence of scars distracts the viewer's gaze away from the patient's face (26, 27). Because of the above considerations, surgeons may prefer to discuss the choice of operation with each patient individually rather than assuming that all patients will prefer remote-access thyroidectomy. An important finding and strength of the current study is that counseling the patients regarding the operation significantly changed their preferences. Weighing the improved cosmesis against the complications preoperatively is vital in decision-making to avoid persuading patients toward scarless thyroidectomy and harboring an increased complication rate (28). Because of that, our findings on decision change following a slideshow are important for both surgeons and patients to avoid conflicts after the operation. Qui et al. (29) conducted a pilot study in China including 100 real patients and found that Saudi medical students were more concerned than Chinese patients about scar perception, body image, pain, and cost. Qui et al. assessed the choice between scarless neck surgery and conventional thyroidectomy in a real-world setting. However, the patients’ decisions may be biased because their choice was assessed immediately after the consultation. In addition, previous suggestions by surgeons might affect the patients' choices. In the present study, we included medical students, 5.6% of whom had undergone a previous conventional thyroidectomy, which represents a limitation. Nevertheless, we assessed their postoperative choice at 2 weeks to allow for brainstorming for a more realistic decision. Our finding significantly challenged routine surgical practice because extensive counseling significantly changed the participants' ideas and choices regarding thyroidectomy.

### Study limitations

This study targeted medical students (5.6% of whom have undergone previous thyroid surgery) at a single university. In addition, the younger age group may not be representative of a real-world setting. Therefore, the current findings cannot be generalized to the entire Kingdom of Saudi Arabia.

## Conclusion

Medical students at the University of Tabuk showed unsatisfactory knowledge about transoral thyroidectomy vestibular approach thyroidectomy, and other remote-access endoscopic thyroidectomies. This is why they chose the remote approach for thyroid removal and significantly changed their minds after 2 weeks following a slideshow and discussion with the authors. The main concerns were scar appearance, postoperative pain, and surgical complications in general. Real-world studies adopting the same approach and comparing it with the current surgical practice are highly recommended.

## Data Availability

The raw data supporting the conclusions of this article will be made available by the authors without undue reservation.

## References

[1] Hegedüs L. Clinical practice. The thyroid nodule. N Engl J Med. (2004) 351(17):1764–71. 10.1056/NEJMcp031436 15496625

[2] Dralle H Machens A Thanh PN. Minimally invasive compared with conventional thyroidectomy for nodular goitre. Best Pract Res Clin Endocrinol Metab. (2014) 28(4):589–99. 10.1016/j.beem.2013.12.002 25047208

[3] Noel CW Griffiths R Siu J Forner D Urbach D Freeman J A population-based analysis of outpatient thyroidectomy: safe and under-utilized. Laryngoscope. (2021) 131(11):2625–33. 10.1002/lary.29816 34378810

[4] Fernandez-Ranvier G Meknat A Guevara DE Inabnet WB 3rd. Transoral endoscopic thyroidectomy vestibular approach. JSLS. (2019) 23(4):e2019.00036. 10.4293/JSLS.2019.00036 31719772 PMC6830499

[5] Anuwong A. Transoral endoscopic thyroidectomy vestibular approach: a series of the first 60 human cases. World J Surg. (2016) 40(3):491–7. 10.1007/s00268-015-3320-1 26546193

[6] Anuwong A Kim HY Dionigi G. Transoral endoscopic thyroidectomy using vestibular approach: updates and evidences. Gland Surg. (2017) 6(3):277–84. 10.21037/gs.2017.03.16 28713700 PMC5503927

[7] Richmon JD Kim HY. Transoral robotic thyroidectomy (TORT): procedures and outcomes. Gland Surg. (2017) 6(3):285–9. 10.21037/gs.2017.05.05 28713701 PMC5503926

[8] Sephton BM. Extracervical approaches to thyroid surgery: evolution and review. Minim Invasive Surg. (2019) 2019:1–14. 10.1155/2019/5961690 31531238 PMC6719267

[9] Alsafran S Quttaineh D Albloushi D Al Safi S Alfawaz A Alyatama K Trans-oral endoscopic endocrine surgery vestibular approach: pioneering the technique in the gulf cooperation council countries—a case series. Ann Med Surg (Lond). (2021) 72:103114. 10.1016/j.amsu.2021.103114 34917349 PMC8646119

[10] Pai VM Muthukumar P Prathap A Leo J Rekha A. Transoral endoscopic thyroidectomy: a case report. Int J Surg Case Rep. (2015) 12:99–101. 10.1016/j.ijscr.2015.04.010 26048629 PMC4485686

[11] Materazzi G Papini P Fregoli L Morganti R De Palma A Ambrosini CE The learning curve on robot-assisted transaxillary thyroidectomy performed by a single endocrine surgeon in a third-level institution in Europe: a cumulative sum (CUSUM) analysis. Updates Surg. (2023) 75(6):1653–60. 10.1007/s13304-023-01619-z 37531041 PMC10435399

[12] Kandil E Akkera M Shalaby H Munshi R Attia A Elnahla A A single surgeon’s 10-year experience in remote-access thyroid and parathyroid surgery. Am Surg. (2021) 87(4):638–44. 10.1177/0003134820950300 33142070

[13] Al Bisher HM Khidr AM Alkhudair BH Alammadi FS Ibrahim AH. Transoral endoscopic thyroidectomy via vestibular approach: first case in Saudi Arabia. Int J Surg Case Rep. (2020) 70:75–7. 10.1016/j.ijscr.2020.04.014 32413772 PMC7226639

[14] Zhao X Bie F Luo C Zhang JE. Distress, illness perception and coping style among thyroid cancer patients after thyroidectomy: a cross-sectional study. Eur J Oncol Nurs. (2024) 69:102517. 10.1016/j.ejon.2024.102517 38340645

[15] Park SK Olweny EO Best SL Tracy CR Mir SA Cadeddu JA. Patient-reported body image and cosmesis outcomes following kidney surgery: comparison of laparoendoscopic single-site, laparoscopic, and open surgery. Eur Urol. (2011) 60(5):1097–104. 10.1016/j.eururo.2011.08.007 21856076

[16] Lang BH Wong CK Tsang JS Wong KP Wan KY. A systematic review and meta-analysis comparing surgically-related complications between robotic-assisted thyroidectomy and conventional open thyroidectomy. Ann Surg Oncol. (2014) 21(3):850–61. 10.1245/s10434-013-3406-7 24271160

[17] Billmann F Bokor-Billmann T Voigt J Kiffner E. Effects of a cost-effective surgical workflow on cosmesis and patient’s satisfaction in open thyroid surgery. Int J Surg. (2013) 11(1):31–6. 10.1016/j.ijsu.2012.11.004 23164990

[18] Calculator.Net. Sample size calculator. Find out the sample size. Available online at: https://www.calculator.net/sample-size-calculator.html?type=1&cl=95&ci=5&pp=50&ps=340&x=79&y=26 (Accessed February 1, 2024).

[19] Coorough NE Schneider DF Rosen MW Sippel RS Chen H Schwarze ML A survey of preferences regarding surgical approach to thyroid surgery. World J Surg. (2014) 38(3):696–703. 10.1007/s00268-013-2405-y 24366272 PMC3936669

[20] Wu V Samargandy S Philteos J Pasternak JD de Almeida JR Monteiro E. Evaluation of preference and utility measures for transoral thyroidectomy. Ann Otol Rhinol Laryngol. (2023) 132(4):381–6. 10.1177/00034894221094950 35503808 PMC9989232

[21] Linos D Kiriakopoulos A Petralias A. Patient attitudes toward transaxillary robot-assisted thyroidectomy. World J Surg. (2013) 37(8):1959–65. 10.1007/s00268-013-2090-x 23665817

[22] Best AR Shipchandler TZ Cordes SR. Midcervical scar satisfaction in thyroidectomy patients. Laryngoscope. (2017) 127(5):1247–52. 10.1002/lary.26177 27519726

[23] Toll EC Loizou P Davis CR Porter GC Pothier DD. Scars and satisfaction: do smaller scars improve patient-reported outcome? Eur Arch Otorhinolaryngol. (2012) 269(1):309–13. 10.1007/s00405-011-1613-z 21544658

[24] Arora A Swords C Garas G Chaidas K Prichard A Budge J The perception of scar cosmesis following thyroid and parathyroid surgery: a prospective cohort study. Int J Surg. (2016) 25:38–43. 10.1016/j.ijsu.2015.11.021 26602967

[25] Sukpanich R Sanglestsawai S Seib CD Gosnell JE Shen WT Roman SA The influence of cosmetic concerns on patient preferences for approaches to thyroid lobectomy: a discrete choice experiment. Thyroid. (2020) 30(9):1306–13. 10.1089/thy.2019.0821 32204688

[26] Juarez MC Ishii L Nellis JC Bater K Huynh PP Fung N Objectively measuring social attention of thyroid neck scars and transoral surgery using eye tracking. Laryngoscope. (2019) 129(12):2789–94. 10.1002/lary.27933 30900247

[27] Rajakumar C Doyle PC Brandt MG Moore CC Nichols A Franklin JH A paired comparison analysis of third-party rater thyroidectomy scar preference. J Laryngol Otol. (2017) 131(1):13–8. 10.1017/S002221511600952X 27917727

[28] Gutknecht S Kaderli R Businger A. Perception of semiquantitative terms in surgery. Ann Surg. (2012) 255(3):589–94. 10.1097/SLA.0b013e31824531ab 22281735

[29] Qiu TY Lau J Wong O Oh HB Boon TW Parameswaran R Preoperative scar perception study comparing ‘scarless’ in the neck endoscopic thyroidectomy with open thyroidectomy: a cross-sectional study. Ann R Coll Surg Engl. (2020) 102(9):737–43. 10.1308/rcsann.2020.0174 32820638 PMC7591632

